# Supplementary material for: The Utility of Virtual Patient Simulations for Clinical Reasoning Education
Source: Int J Environ Res Public Health. 2020 Jul 24;17(15):5325. doi: 10.3390/ijerph17155325 (PMC7432110; doi:10.3390/ijerph17155325)
Supplement: Supplementary file 1 [file ijerph-17-05325-s001.pdf]

International Journal of
Environmental Research and Public Health

List of 20 MCQs:

No. 1: Clinical reasoning

Management of altered mental status

A 60-year-old man has been transported to the emergency room of the hospital with altered mental status. His present illness and past medical history are unknown. Which of the following differential diagnoses should be considered first?

1. Cerebral infarction
2. Cerebral hemorrhage
3. Hypoxemia
4. Hypoglycemia
5. Shock

No. 2: Knowledge

Electrocardiogram and syncope

Which arrhythmia is most likely to cause fainting?

1. Complete right leg block
2. Complete left leg block
3. Complete atrioventricular blocks
4. Wenckebach-type 2-degree atrioventricular block
5. Premature ventricular contraction

No. 3: Knowledge

Type of hormone secreted during hypoglycemia

Which hormones do not increase blood levels during hypoglycemia?

1. Glucagon
2. Adrenaline
3. Cortisol
4. Growth hormone
5. Parathormone

No. 4: Knowledge

Referred pain due to acute coronary syndrome

Which of the following is not usually seen in acute coronary syndrome?

1. Nausea
2. Left shoulder pain
3. Auricular pain
4. Fainting
5. Cold sweat

No. 5: Clinical reasoning

Management of altered mental status

You want to do a differential for altered mental status and syncope.

Which of the following describes the characteristics and symptoms of syncope correctly?

1. Fever
2. Recovery in several minutes
3. Elevated blood pressure
4. Facial flushing
5. Hypoglycemia

No. 6: Knowledge

Pathophysiology of pulmonary failure

Which of the following is not a cause of acute respiratory failure?

1. Sepsis
2. Pulmonary contusion
3. Gastric fluid aspiration
4. Hyperventilation syndrome
5. Pulmonary thromboembolism

No. 7: Knowledge

Electrocardiogram (EKG) of ST elevation

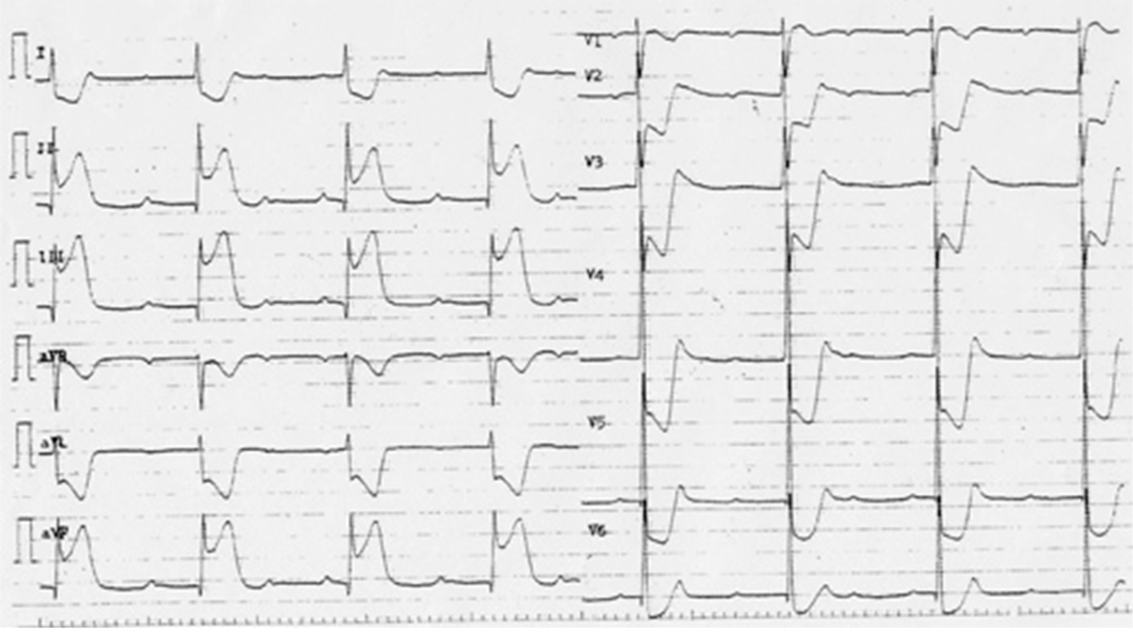

EKG is shown. What is the diagnosis?

1. Acute myocarditis
2. Myocardial infarction (anterior wall)
3. Severe pulmonary embolism
4. Abdominal aortic dissection
5. Myocardial infarction (posterior wall)

No. 8: Clinical reasoning

Management of altered mental status

An 80-year-old woman was admitted for hip replacement surgery. Her mental status changed on the fourth postoperative day. You examined the patient. Which finding is not an indicator that the patient's condition is urgent?

1. Cold sweat
2. Shivering
3. CRP 12 mg/dL
4. Blood pressure 82/42 mmHg, pulse 120/min
5. Respiratory rate > 24 /min

No. 9: Knowledge

Anatomy of aortic dissection

Which of the following is not observed in acute aortic dissection?

1. Paraplegia
2. Loss of consciousness
3. Decreased urine output

4. Raynaud phenomenon
5. Differences in the left and right upper extremity blood pressures

No. 10: Clinical reasoning

Management of each type of shock

A 42-year-old woman complained of fever and headache yesterday. She is a known case of diabetes mellitus. Assessment of vital signs revealed Glasgow Coma Scale (GCS) 15, body temperature (BT) 39.0°C, blood pressure (BP) 98/42mmHg, heart rate (HR) 130/min, respiratory rate (RR) 28/min, and SpO2 98%

Which of the following is not the first response you should consider with this patient?

1. Blood sugar test
2. Evaluation of meningeal signs
3. Brain MRI
4. Massive fluid infusion
5. Blood culture

No. 11: Knowledge

Contrast CT of aortic dissection

A 75-year-old woman presented to the hospital with sudden chest pain. What is the diagnosis?

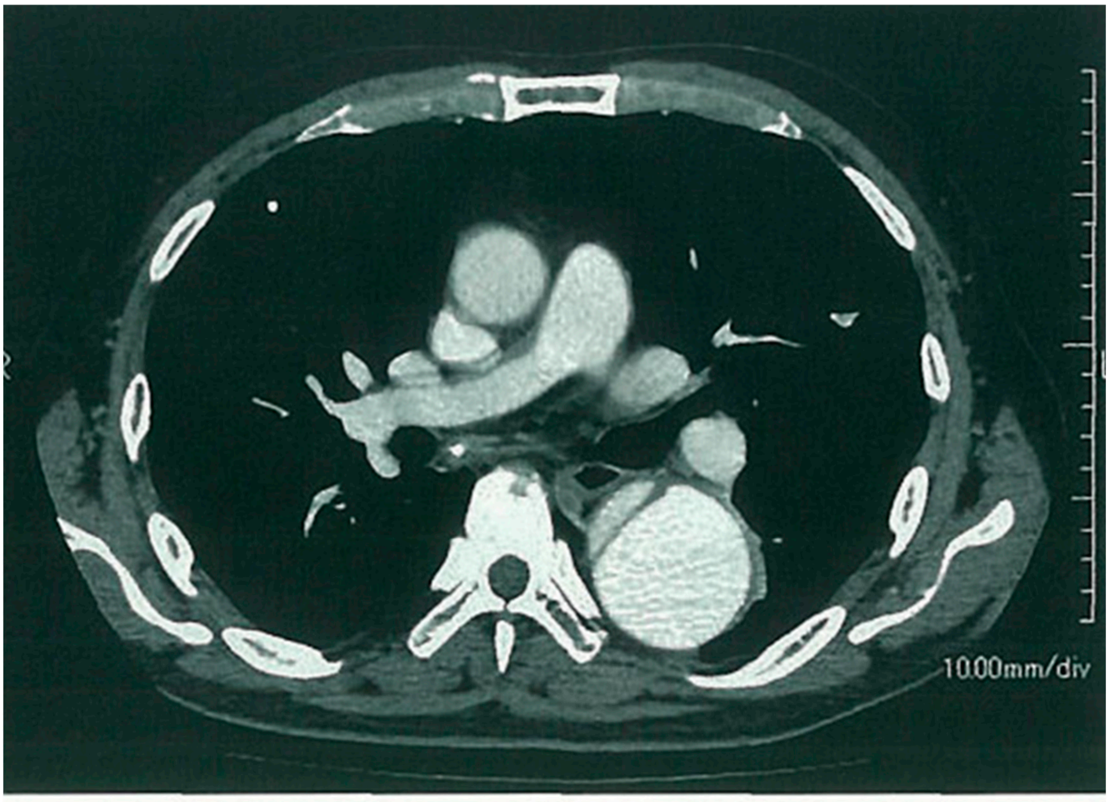

1. Myocardial infarction
2. Pulmonary embolism
3. Aortic dissection
4. Tension pneumothorax
5. Airway obstruction

No. 12: Clinical reasoning

Management of altered mental status

A 68-year-old man presented to the Internal Medicine outpatient department with chief complaints of fever and respiratory distress. His vital signs were as follows: body temperature 39.2

°C, HR 110/min, BP 98/42 mmHg, RR 24/min, and SpO2 91%. Which of the following is the most likely combination of diagnosis and treatment?

1. Sepsis and massive fluid drip infusion
2. Massive bleeding and administration of steroids
3. Tension pneumothorax and positive pressure ventilation
4. Pulmonary embolism and digitalis
5. Hyperkalemia and magnesium sulfate

No. 13: Knowledge

Jugular venous pressure

Which of the following is a condition that presents with shock and is associated with high central venous pressure?

1. Anaphylactic shock
2. Tension pneumothorax
3. Sepsis
4. Ruptured esophageal varices
5. Abdominal aortic aneurysm rupture

No. 14: Clinical reasoning

Management of altered mental status

A 42-year-old man with type 1 diabetes was transported to the emergency room due to symptoms of cold sweat, palpitations, and disorientation. Left-sided paralysis was observed on admission of the patient. Which of the following is the most appropriate response?

1. Rapid intravenous infusion of high concentration (50%) of glucose
2. Start a 5% dextrose drip
3. Emergency MRI to rule out stroke
4. Contrast-enhanced CT for suspected aortic dissection
5. Electrocardiogram

No. 15: Clinical reasoning

Management of altered mental status

An elderly patient, who did not have any information about his/her history, situation, family history, or drug history, was transported to your hospital. Which of the following is the appropriate initial approach to such a patient who presents with altered mental status?

1. Deny hypoglycemia
2. Head CT for evaluation of cerebral hemorrhage
3. Head MRI
4. Rapid lumbar puncture
5. Blood culture

No. 16: Knowledge

Chest radiograph of heart failure

A 65-year-old man presented to the hospital with wheezing. Chest X-ray is shown.

Which is the most likely diagnosis?

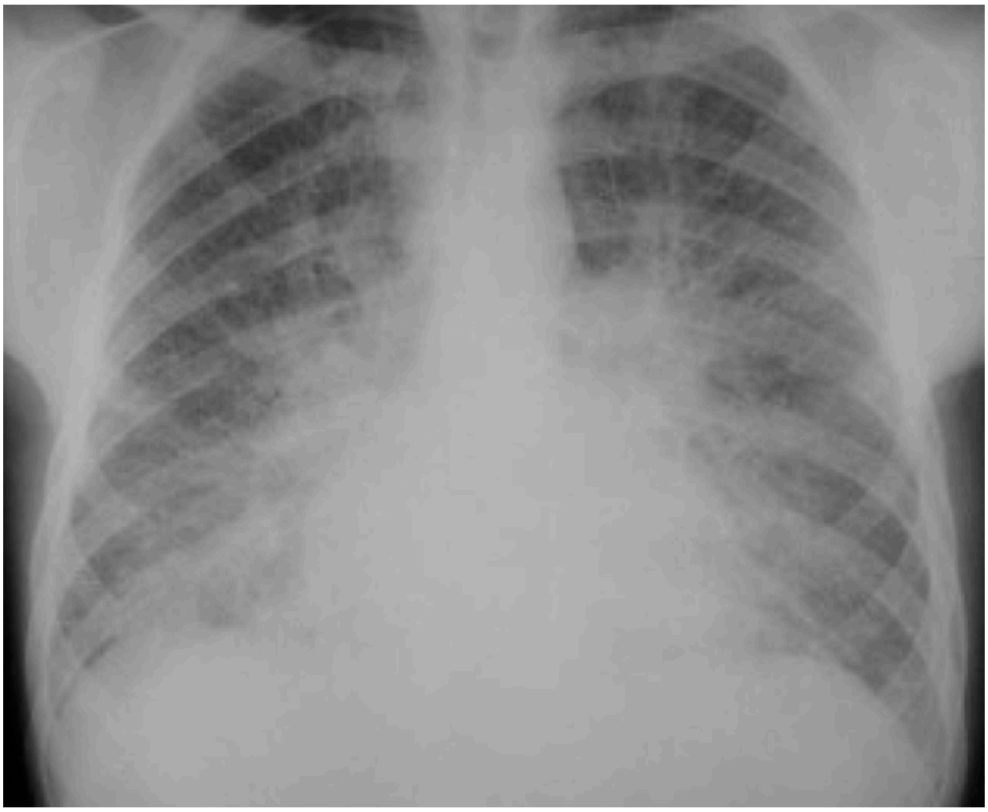

1. Atypical pneumonia
2. Acute heart failure
3. Pulmonary embolism
4. Pneumococcal pneumonia
5. Interstitial pneumonia

No. 17: Clinical reasoning

Management of altered mental status

A 32-year-old woman was transported to the emergency room due to sudden loss of consciousness. No symptoms were observed on arrival. Which of the following is the most important response to rule the differential diagnosis of syncope?

1. Rapid head CT
2. MRI in consideration of a transient ischemic attack
3. Detailed medical history before and after the symptoms
4. ECG
5. Assessment of anemia by blood test

No. 18: Knowledge

Symptoms of hypoglycemia

Which of the following symptoms are not commonly seen in patients with hypoglycemia?

1. Hemiplegia
2. Tachycardia
3. Cold sweating
4. Low blood pressure
5. Altered mental status

No. 19: Clinical reasoning

Management of altered mental status

An unconscious 72-year-old man was brought to the emergency room. He had cough with sputum for a week and gradually lost energy. A family member had called out to him in the morning

and transported him to the hospital because he was unresponsive. His vital signs were as follows: GCS, 11(E3V4M5); temperature, 38.6°C; HR 112/min; blood pressure, 80/60 mmHg; respiratory rate, 30 breaths per minute; and SpO_2, 93% (4 L of oxygen). Lung sounds revealed coarse crackles in the right dorsal region. Which of the following actions should be taken first?

1. Penicillin antibiotic drip
2. Intravenous adrenaline
3. Intravenous digoxin
4. Rapid infusion of saline
5. Drip infusion of steroids

No. 20: Clinical reasoning

Management of chest pain

A middle-aged diabetic man was brought to the emergency room with acute sudden onset chest pain. Pulse rate was 120/minute. Blood pressure was 160/94 mmHg and BT was 36.1°C. He complained of discomfort in his back as well. He had cold sensation and prominent sweating in the extremities, but there was no edema. Which of the following tests should not be performed first?

1. Blood culture
2. ECG
3. CT of the chest
4. Arterial blood fluid gas
5. Blood sugar

© 2020 by the authors. Licensee MDPI, Basel, Switzerland. This article is an open access article distributed under the terms and conditions of the Creative Commons Attribution (CC BY) license (http://creativecommons.org/licenses/by/4.0/).
